# (Acridine-κ*N*)(dimethyl sulfoxide-κ*S*)diiodidoplatinum(II)

**DOI:** 10.1107/S1600536810031387

**Published:** 2010-08-11

**Authors:** Kwang Ha

**Affiliations:** a School of Applied Chemical Engineering, The Research Institute of Catalysis, Chonnam National University, Gwangju 500-757, Republic of Korea

## Abstract

In the title complex, [PtI_2_(C_13_H_9_N)(C_2_H_6_OS)], the Pt^II^ atom is four-coordinated in an essentially square-planar environment defined by the N atom of the acridine ligand, the S atom of dimethyl sulfoxide, and two iodide ions. The dihedral angle between the nearly planar PtI_2_NS unit [maximum deviation = 0.083 (2) Å] and the acridine ligand [maximum deviation = 0.038 (6) Å] is 89.29 (7)°. In the crystal structure, the complex mol­ecules are arranged in a V-shaped packing pattern along the *c* axis and linked by inter­molecular C—H⋯O contacts into supra­molecular chains. There are also several inter­molecular π–π inter­actions between the six-membered rings, with a shortest ring centroid–centroid distance of 3.804 (5) Å.

## Related literature

For the crystal structures of [PtCl_2_(acr)_2_] (acr = acridine) and [PtCl(pic)(DMSO)] (pic = pyridine-2-carboxyl­ate, DMSO = dimethyl sulfoxide), see: Ha (2010*a* ▶,*b* ▶).
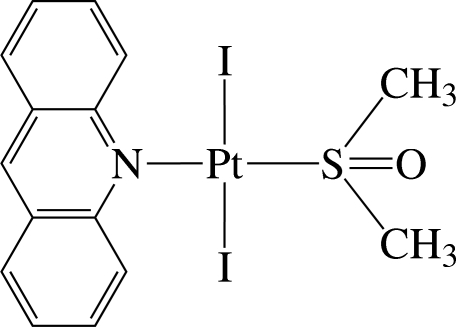


## Experimental

### 

#### Crystal data

[PtI_2_(C_13_H_9_N)(C_2_H_6_OS)]
*M* *_r_* = 706.23
Monoclinic, 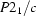

*a* = 8.4800 (6) Å
*b* = 23.8181 (17) Å
*c* = 9.9036 (7) Å
β = 114.492 (1)°
*V* = 1820.3 (2) Å^3^
*Z* = 4
Mo *K*α radiation
μ = 11.21 mm^−1^
*T* = 200 K
0.20 × 0.19 × 0.06 mm

#### Data collection

Bruker SMART 1000 CCD diffractometer
Absorption correction: multi-scan (*SADABS*; Bruker, 2000 ▶) *T*_min_ = 0.468, *T*_max_ = 1.000
11217 measured reflections
3573 independent reflections
2809 reflections with *I* > 2σ(*I*)
*R*_int_ = 0.051

#### Refinement

*R*[*F* ^2^ > 2σ(*F* ^2^)] = 0.035
*wR*(*F* ^2^) = 0.083
*S* = 1.03
3573 reflections
192 parameters
H-atom parameters constrained
Δρ_max_ = 2.10 e Å^−3^
Δρ_min_ = −1.16 e Å^−3^

### 

Data collection: *SMART* (Bruker, 2000 ▶); cell refinement: *SAINT* (Bruker, 2000 ▶); data reduction: *SAINT*; program(s) used to solve structure: *SHELXS97* (Sheldrick, 2008 ▶); program(s) used to refine structure: *SHELXL97* (Sheldrick, 2008 ▶); molecular graphics: *ORTEP-3* (Farrugia, 1997 ▶) and *PLATON* (Spek, 2009 ▶); software used to prepare material for publication: *SHELXL97*.

## Supplementary Material

Crystal structure: contains datablocks global, I. DOI: 10.1107/S1600536810031387/tk2697sup1.cif

Structure factors: contains datablocks I. DOI: 10.1107/S1600536810031387/tk2697Isup2.hkl

Additional supplementary materials:  crystallographic information; 3D view; checkCIF report

## Figures and Tables

**Table 1:** Selected bond lengths (Å)

Pt1—N1	2.083 (6)
Pt1—S1	2.222 (2)
Pt1—I1	2.6082 (6)
Pt1—I2	2.6160 (6)

**Table 2:** Hydrogen-bond geometry (Å, °)

*D*—H⋯*A*	*D*—H	H⋯*A*	*D*⋯*A*	*D*—H⋯*A*
C14—H14*B*⋯O1^i^	0.98	2.36	3.209 (10)	145
C15—H15*B*⋯O1^i^	0.98	2.37	3.241 (10)	147

Symmetry code: (i) 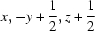.

**Fig. 1.**
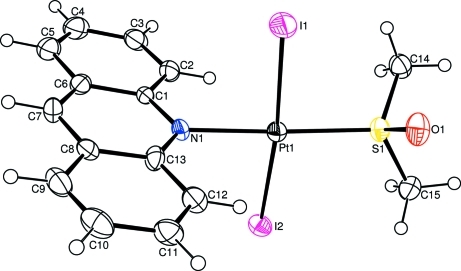
The molecular structure of the title complex showing atom labelling. Displacement ellipsoids drawn at the 40% probability level for non-H atoms.

**Fig. 2.**
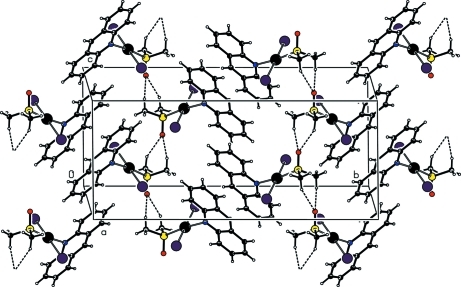
View of the unit-cell contents of the title complex. Hydrogen-bond interactions are drawn with dashed lines.

## supplementary crystallographic information

### Comment

Single crystals of the title complex, [PtI_2_(C_13_H_9_N)(C_2_H_6_OS)] (where C_13_H_9_N is acridine (acr) and C_2_H_6_OS is dimethyl sulfoxide (DMSO)), were unexpectedly obtained from a DMSO solution of the dark-yellow reaction product [PtI_2_(acr)_2_] held at 353 K. It seems that an acridine ligand of the complex [PtI_2_(acr)_2_] was replaced by a DMSO molecule during crystallization, whereas the analogous Pt complex [PtCl_2_(acr)_2_] crystallized without substitution in a DMSO solution at 353 K (Ha, 2010*a*). In the title complex, the Pt^II^ atom is four-coordinated in an essentially square-planar environment defined by the N atom of the acridine ligand, the S atom of the dimethyl sulfoxide molecule and two iodide ions (Table 1 and Fig. 1). The dihedral angle between the nearly planar PtI_2_NS moiety and acridine ligand is 89.29 (7)°. The I atoms are in *trans* conformation with respect to each other (<I1—Pt1—I2 = 174.03 (2)°) and almost perpendicular to the acridine ligand, with N1—Pt1—I1/2 bond angles of 85.75 (16)° and 89.21 (16)°. The Pt—S bond length (2.222 (2) Å) is comparable to those observed in the Pt-DMSO complex [PtCl(pic)(DMSO)] (2.202 (2) Å, where pic is pyridine-2-carboxylate (Ha, 2010*b*). In the crystal structure, the complexes are arranged in a V-shaped packing pattern along the *c* axis and linked by intermolecular C—H···O contacts into one-dimensional supramolecular chains (Table 2 and Fig. 2). There are also numerous intermolecular π-π interactions between six-membered rings, with the shortest ring centroid-centroid distance being 3.804 (5) Å.

### Experimental

To a solution of K_2_PtCl_4_ (0.2017 g, 0.486 mmol) and KI (0.6410 g, 3.861 mmol) in H_2_O (30 ml) was added acridine (0.1903 g, 1.062 mmol) followed by refluxing for 3 h. The precipitate was then separated by filtration, washed with H_2_O and EtOH, and dried under vacuum, to give a dark-yellow powder (0.2772 g). Crystals suitable for X-ray analysis were obtained by slow evaporation from a dimethyl sulfoxide at 353 K.

### Refinement

H atoms were positioned geometrically and allowed to ride on their respective parent atoms [C—H = 0.95 Å (CH) or 0.98 Å (CH_3_) and *U*_iso_(H) = 1.2*U*_eq_(C) or 1.5*U*_eq_(methyl-C)]. The highest peak (2.10 e Å^-3^) and the deepest hole (-1.16 e Å^-3^) in the difference Fourier map were located 1.05 and 1.64 Å from the atoms Pt1 and I2, respectively.

### Crystal data

**Table:**

[PtI_2_(C_13_H_9_N)(C_2_H_6_OS)]	*F*(000) = 1280
*M**_r_* = 706.23	*D*_x_ = 2.577 Mg m^−^^3^
Monoclinic, *P*2_1_/*c*	Mo *K*α radiation, λ = 0.71073 Å
Hall symbol: -P 2ybc	Cell parameters from 5088 reflections
*a* = 8.4800 (6) Å	θ = 2.6–26.0°
*b* = 23.8181 (17) Å	µ = 11.21 mm^−^^1^
*c* = 9.9036 (7) Å	*T* = 200 K
β = 114.492 (1)°	Block, yellow
*V* = 1820.3 (2) Å^3^	0.20 × 0.19 × 0.06 mm
*Z* = 4	

### Data collection

**Table:**

Bruker SMART 1000 CCD diffractometer	3573 independent reflections
Radiation source: fine-focus sealed tube	2809 reflections with *I* > 2σ(*I*)
graphite	*R*_int_ = 0.051
φ and ω scans	θ_max_ = 26.0°, θ_min_ = 2.4°
Absorption correction: multi-scan (*SADABS*; Bruker, 2000)	*h* = −10→7
*T*_min_ = 0.468, *T*_max_ = 1.000	*k* = −28→29
11217 measured reflections	*l* = −12→12

### Refinement

**Table:**

Refinement on *F*^2^	Primary atom site location: structure-invariant direct methods
Least-squares matrix: full	Secondary atom site location: difference Fourier map
*R*[*F*^2^ > 2σ(*F*^2^)] = 0.035	Hydrogen site location: inferred from neighbouring sites
*wR*(*F*^2^) = 0.083	H-atom parameters constrained
*S* = 1.03	*w* = 1/[σ^2^(*F*_o_^2^) + (0.0324*P*)^2^] where *P* = (*F*_o_^2^ + 2*F*_c_^2^)/3
3573 reflections	(Δ/σ)_max_ < 0.001
192 parameters	Δρ_max_ = 2.10 e Å^−^^3^
0 restraints	Δρ_min_ = −1.16 e Å^−^^3^

### Special details

**Table:**

Geometry. All e.s.d.'s (except the e.s.d. in the dihedral angle between two l.s. planes) are estimated using the full covariance matrix. The cell e.s.d.'s are taken into account individually in the estimation of e.s.d.'s in distances, angles and torsion angles; correlations between e.s.d.'s in cell parameters are only used when they are defined by crystal symmetry. An approximate (isotropic) treatment of cell e.s.d.'s is used for estimating e.s.d.'s involving l.s. planes.
Refinement. Refinement of *F*^2^ against ALL reflections. The weighted *R*-factor *wR* and goodness of fit *S* are based on *F*^2^, conventional *R*-factors *R* are based on *F*, with *F* set to zero for negative *F*^2^. The threshold expression of *F*^2^ > σ(*F*^2^) is used only for calculating *R*-factors(gt) *etc*. and is not relevant to the choice of reflections for refinement. *R*-factors based on *F*^2^ are statistically about twice as large as those based on *F*, and *R*- factors based on ALL data will be even larger.

### Fractional atomic coordinates and isotropic or equivalent isotropic displacement parameters (Å^2^)

**Table:**

	*x*	*y*	*z*	*U*_iso_*/*U*_eq_	
Pt1	0.22004 (4)	0.152591 (13)	0.21512 (3)	0.02504 (11)	
I1	0.44358 (8)	0.19167 (3)	0.12073 (8)	0.04961 (19)	
I2	0.02342 (7)	0.10938 (2)	0.33373 (6)	0.03254 (15)	
S1	0.0408 (2)	0.22130 (9)	0.0922 (2)	0.0282 (4)	
O1	−0.0003 (7)	0.2220 (3)	−0.0675 (6)	0.0410 (15)	
N1	0.3965 (8)	0.0876 (3)	0.3075 (7)	0.0282 (15)	
C1	0.5309 (9)	0.0945 (3)	0.4428 (8)	0.0247 (17)	
C2	0.5382 (11)	0.1408 (4)	0.5345 (9)	0.036 (2)	
H2	0.4474	0.1678	0.5020	0.043*	
C3	0.6736 (11)	0.1471 (4)	0.6683 (10)	0.039 (2)	
H3	0.6754	0.1780	0.7295	0.047*	
C4	0.8130 (11)	0.1081 (4)	0.7186 (10)	0.046 (3)	
H4	0.9081	0.1136	0.8118	0.056*	
C5	0.8103 (10)	0.0635 (4)	0.6344 (10)	0.042 (2)	
H5	0.9044	0.0378	0.6686	0.050*	
C6	0.6695 (10)	0.0543 (4)	0.4955 (9)	0.034 (2)	
C7	0.6615 (10)	0.0093 (4)	0.4048 (10)	0.039 (2)	
H7	0.7548	−0.0167	0.4367	0.047*	
C8	0.5221 (10)	0.0007 (3)	0.2686 (9)	0.0313 (19)	
C9	0.5072 (11)	−0.0455 (4)	0.1740 (11)	0.042 (2)	
H9	0.5958	−0.0732	0.2045	0.050*	
C10	0.3711 (12)	−0.0513 (4)	0.0421 (10)	0.045 (2)	
H10	0.3649	−0.0821	−0.0207	0.054*	
C11	0.2376 (11)	−0.0108 (4)	−0.0020 (9)	0.037 (2)	
H11	0.1403	−0.0152	−0.0943	0.044*	
C12	0.2453 (10)	0.0344 (4)	0.0852 (9)	0.035 (2)	
H12	0.1538	0.0610	0.0529	0.042*	
C13	0.3888 (10)	0.0418 (3)	0.2234 (9)	0.0296 (18)	
C14	0.1268 (10)	0.2874 (4)	0.1699 (9)	0.036 (2)	
H14A	0.2394	0.2928	0.1661	0.055*	
H14B	0.1415	0.2887	0.2734	0.055*	
H14C	0.0471	0.3172	0.1134	0.055*	
C15	−0.1582 (10)	0.2226 (4)	0.1040 (9)	0.038 (2)	
H15A	−0.2275	0.2540	0.0453	0.057*	
H15B	−0.1395	0.2272	0.2079	0.057*	
H15C	−0.2197	0.1872	0.0655	0.057*	

### Atomic displacement parameters (Å^2^)

**Table:**

	*U*^11^	*U*^22^	*U*^33^	*U*^12^	*U*^13^	*U*^23^
Pt1	0.02442 (17)	0.0238 (2)	0.02705 (18)	0.00111 (12)	0.01078 (13)	0.00131 (13)
I1	0.0400 (4)	0.0479 (4)	0.0741 (5)	0.0065 (3)	0.0368 (3)	0.0194 (3)
I2	0.0375 (3)	0.0295 (3)	0.0353 (3)	−0.0020 (2)	0.0198 (2)	0.0003 (2)
S1	0.0316 (11)	0.0290 (12)	0.0239 (10)	0.0046 (8)	0.0113 (8)	0.0022 (8)
O1	0.050 (4)	0.052 (4)	0.020 (3)	0.011 (3)	0.014 (3)	0.005 (3)
N1	0.028 (4)	0.019 (4)	0.039 (4)	0.000 (3)	0.015 (3)	0.003 (3)
C1	0.017 (4)	0.021 (5)	0.029 (4)	0.002 (3)	0.002 (3)	0.009 (3)
C2	0.039 (5)	0.028 (5)	0.035 (5)	0.000 (4)	0.009 (4)	0.006 (4)
C3	0.042 (5)	0.034 (6)	0.032 (5)	−0.011 (4)	0.007 (4)	0.004 (4)
C4	0.035 (5)	0.054 (7)	0.033 (5)	−0.012 (5)	−0.003 (4)	0.013 (5)
C5	0.027 (5)	0.047 (7)	0.040 (5)	−0.001 (4)	0.002 (4)	0.013 (5)
C6	0.031 (5)	0.037 (6)	0.037 (5)	−0.005 (4)	0.016 (4)	0.008 (4)
C7	0.034 (5)	0.033 (6)	0.053 (6)	0.009 (4)	0.020 (4)	0.016 (4)
C8	0.028 (4)	0.021 (5)	0.048 (5)	0.000 (3)	0.018 (4)	0.008 (4)
C9	0.047 (6)	0.028 (6)	0.062 (6)	0.004 (4)	0.034 (5)	0.006 (5)
C10	0.070 (7)	0.027 (6)	0.051 (6)	−0.008 (5)	0.037 (5)	−0.013 (4)
C11	0.036 (5)	0.037 (6)	0.033 (5)	−0.003 (4)	0.011 (4)	−0.002 (4)
C12	0.035 (5)	0.028 (5)	0.042 (5)	−0.001 (4)	0.015 (4)	0.001 (4)
C13	0.035 (5)	0.024 (5)	0.035 (5)	0.007 (4)	0.020 (4)	0.006 (4)
C14	0.042 (5)	0.029 (6)	0.035 (5)	0.002 (4)	0.013 (4)	0.003 (4)
C15	0.031 (5)	0.034 (6)	0.043 (5)	0.006 (4)	0.009 (4)	0.006 (4)

### Geometric parameters (Å, °)

**Table:**

Pt1—N1	2.083 (6)	C6—C7	1.381 (12)
Pt1—S1	2.222 (2)	C7—C8	1.392 (11)
Pt1—I1	2.6082 (6)	C7—H7	0.9500
Pt1—I2	2.6160 (6)	C8—C9	1.417 (12)
S1—O1	1.472 (5)	C8—C13	1.420 (10)
S1—C15	1.740 (8)	C9—C10	1.344 (13)
S1—C14	1.771 (9)	C9—H9	0.9500
N1—C13	1.358 (10)	C10—C11	1.410 (12)
N1—C1	1.362 (9)	C10—H10	0.9500
C1—C2	1.414 (11)	C11—C12	1.364 (11)
C1—C6	1.435 (11)	C11—H11	0.9500
C2—C3	1.355 (11)	C12—C13	1.415 (11)
C2—H2	0.9500	C12—H12	0.9500
C3—C4	1.420 (12)	C14—H14A	0.9800
C3—H3	0.9500	C14—H14B	0.9800
C4—C5	1.345 (13)	C14—H14C	0.9800
C4—H4	0.9500	C15—H15A	0.9800
C5—C6	1.416 (11)	C15—H15B	0.9800
C5—H5	0.9500	C15—H15C	0.9800
			
N1—Pt1—S1	172.71 (17)	C6—C7—C8	122.5 (8)
N1—Pt1—I1	85.75 (16)	C6—C7—H7	118.7
S1—Pt1—I1	88.55 (5)	C8—C7—H7	118.7
N1—Pt1—I2	89.21 (16)	C7—C8—C9	124.3 (8)
S1—Pt1—I2	96.73 (5)	C7—C8—C13	116.7 (8)
I1—Pt1—I2	174.03 (2)	C9—C8—C13	119.0 (8)
O1—S1—C15	105.5 (4)	C10—C9—C8	121.8 (8)
O1—S1—C14	109.1 (4)	C10—C9—H9	119.1
C15—S1—C14	101.0 (4)	C8—C9—H9	119.1
O1—S1—Pt1	113.6 (3)	C9—C10—C11	119.1 (8)
C15—S1—Pt1	116.0 (3)	C9—C10—H10	120.4
C14—S1—Pt1	110.6 (3)	C11—C10—H10	120.4
C13—N1—C1	120.5 (7)	C12—C11—C10	121.4 (8)
C13—N1—Pt1	119.0 (5)	C12—C11—H11	119.3
C1—N1—Pt1	119.9 (5)	C10—C11—H11	119.3
N1—C1—C2	121.4 (7)	C11—C12—C13	120.4 (8)
N1—C1—C6	120.2 (7)	C11—C12—H12	119.8
C2—C1—C6	118.4 (7)	C13—C12—H12	119.8
C3—C2—C1	120.5 (8)	N1—C13—C12	119.8 (7)
C3—C2—H2	119.7	N1—C13—C8	122.0 (7)
C1—C2—H2	119.7	C12—C13—C8	118.2 (8)
C2—C3—C4	121.0 (9)	S1—C14—H14A	109.5
C2—C3—H3	119.5	S1—C14—H14B	109.5
C4—C3—H3	119.5	H14A—C14—H14B	109.5
C5—C4—C3	120.0 (8)	S1—C14—H14C	109.5
C5—C4—H4	120.0	H14A—C14—H14C	109.5
C3—C4—H4	120.0	H14B—C14—H14C	109.5
C4—C5—C6	121.1 (8)	S1—C15—H15A	109.5
C4—C5—H5	119.5	S1—C15—H15B	109.5
C6—C5—H5	119.5	H15A—C15—H15B	109.5
C7—C6—C5	123.1 (8)	S1—C15—H15C	109.5
C7—C6—C1	117.9 (7)	H15A—C15—H15C	109.5
C5—C6—C1	118.9 (8)	H15B—C15—H15C	109.5
			
I1—Pt1—S1—O1	57.2 (3)	C2—C1—C6—C7	−179.7 (7)
I2—Pt1—S1—O1	−125.6 (3)	N1—C1—C6—C5	177.9 (7)
I1—Pt1—S1—C15	179.8 (3)	C2—C1—C6—C5	−2.1 (11)
I2—Pt1—S1—C15	−3.0 (3)	C5—C6—C7—C8	179.9 (8)
I1—Pt1—S1—C14	−65.9 (3)	C1—C6—C7—C8	−2.6 (12)
I2—Pt1—S1—C14	111.3 (3)	C6—C7—C8—C9	−178.6 (7)
I1—Pt1—N1—C13	−87.4 (5)	C6—C7—C8—C13	2.2 (12)
I2—Pt1—N1—C13	95.8 (5)	C7—C8—C9—C10	−178.7 (8)
I1—Pt1—N1—C1	84.1 (5)	C13—C8—C9—C10	0.6 (12)
I2—Pt1—N1—C1	−92.8 (5)	C8—C9—C10—C11	−1.4 (13)
C13—N1—C1—C2	−177.6 (7)	C9—C10—C11—C12	1.2 (13)
Pt1—N1—C1—C2	11.0 (9)	C10—C11—C12—C13	−0.1 (13)
C13—N1—C1—C6	2.4 (11)	C1—N1—C13—C12	178.0 (7)
Pt1—N1—C1—C6	−169.0 (5)	Pt1—N1—C13—C12	−10.6 (9)
N1—C1—C2—C3	−179.6 (7)	C1—N1—C13—C8	−2.9 (11)
C6—C1—C2—C3	0.4 (12)	Pt1—N1—C13—C8	168.6 (6)
C1—C2—C3—C4	1.3 (13)	C11—C12—C13—N1	178.5 (7)
C2—C3—C4—C5	−1.3 (14)	C11—C12—C13—C8	−0.7 (12)
C3—C4—C5—C6	−0.4 (13)	C7—C8—C13—N1	0.6 (11)
C4—C5—C6—C7	179.6 (8)	C9—C8—C13—N1	−178.7 (7)
C4—C5—C6—C1	2.1 (12)	C7—C8—C13—C12	179.8 (7)
N1—C1—C6—C7	0.3 (11)	C9—C8—C13—C12	0.5 (11)

### Hydrogen-bond geometry (Å, °)

**Table:**

*D*—H···*A*	*D*—H	H···*A*	*D*···*A*	*D*—H···*A*
C14—H14B···O1^i^	0.98	2.36	3.209 (10)	145.
C15—H15B···O1^i^	0.98	2.37	3.241 (10)	147.

Symmetry codes: (i) *x*, −*y*+1/2, *z*+1/2.
